# Open questions: how to get developmental biology into shape?

**DOI:** 10.1186/s12915-019-0636-6

**Published:** 2019-02-22

**Authors:** Timothy E. Saunders, Philip W. Ingham

**Affiliations:** 1 Mechanobiology Institute and Department of Biological Sciences, National University of Singapore, Singapore, Singapore; 2 Lee Kong Chian School of Medicine, Nanyang Technological University, Singapore, Singapore; 3 Institute of Molecular and Cell Biology, A*Star, Singapore, Singapore; 4 Living Systems Institute, University of Exeter, Exeter, UK

## Abstract

Recent technical advances have provided unprecedented insights into the selective deployment of the genome in developing organisms, but how such differential gene expression is used to sculpt the complex shapes and sizes of organs remains unclear. Here, we outline major open questions in organogenesis and suggest how a synthesis between developmental biology and physics can help to address them.

## Why do shape and size matter?

The function of an organ depends critically upon its shape. A stunning illustration of this is the vertebrate inner ear, where the exquisite architecture of the semi-circular canals is essential for balance [1]. The remarkable variety in the shapes and sizes of beaks amongst the different species of Darwin’s finches provides another classic example of how form evolves to be perfectly adapted to function: in each case, the size and shape of the beak reflects food accessibility in different habitats. Although molecular genetic studies have provided some insight into the biochemical pathways that underlie the generation of such morphological diversity, we still lack a deep understanding of the cellular processes that drive morphogenesis.

## How do you know you are the right size?

During growth, organs must integrate information about system size; for example, how do our arms and legs grow to be equal length? Positional information for future organs is provided through spatial signalling pathways, such as morphogen gradients. Morphogens and the downstream gene regulatory networks can generate scaled gene expression boundaries with remarkable precision in the *Drosophila* embryo [2]. However, most embryonic development occurs in growing tissues. Controlling size is a multiscale process: from the nuclear level, where spindle size has to be carefully regulated, to tissue scaling and organism size control. Topological constraints and mechanical inputs may also play a role in size control, as demonstrated by the mechanosensitive Hippo/YAP pathway [3].

Growth is not restricted to developing systems; in a range of organisms organs can regenerate. In zebrafish, the tail can regenerate after injury to almost the exact proportions of the original tail. Internal organs such as the heart can also regenerate and mechanical forces may play an important role in guiding such a process [4]. How organ size is regulated during regeneration is an intriguing open question with clear relevance to both basic researchers and the clinic.

## Can developing systems tell the time?

Precise temporal control of gene expression and cell proliferation and rearrangements is essential during organogenesis. Recently, exciting progress has been made in understanding how timing is regulated in the *Drosophila* wing disc, with new pathways identified that could be playing a role in regulating the temporal trajectory of development. However, we still have very limited understanding of how temporal coordination and growth are interlinked (arguably with the exception of the vertebrate somite clock). Inter-organ communication via hormone secretion has been hypothesised as a regulatory mechanism [5]. An important step is to start recording developmental time with the precision with which we measure position and boundaries in development. Such quantitative data can be used to rigorously test models of temporal control. Ultimately, in order to understand how complex shape emerges, we need to look more carefully at *when* events happen, as well as *where.*

## Is development flat?

No; however, a lot of work in development of tissues focuses on relatively flat, accessible tissues, such as the *Drosophila* wing-disc. With improvements in microscopy, such as light-sheet and structured illumination microscopy, we have the tools to record development at sufficient temporal and spatial resolution to enable in toto exploration of developing tissues. These data enable direct testing of models of morphogenesis, such as in branching morphogenesis of the kidney. Another illustration is imaging of the beating zebrafish heart during development [6], which has helped decipher how the pumping action of the heart feeds back into guiding its morphogenesis. In epithelial tissues, recent work has highlighted how the processes of cell rearrangement and intercalation can occur at both apical and basal surfaces. We see that new biology is emerging as systems are observed at higher spatial and temporal resolutions.

## How are final organ shape and size so reliably formed?

The specification of specific cell fates is not the end of making an organ. How are cell fate maps converted robustly into functioning tissues? The role of genetic feedback mechanisms has been intensively studied, but mechanical feedback also likely plays a crucial role in regulating morphogenesis. A major outstanding question is how mechanical information is reliably interpreted at the cellular level? Possible solutions to these problems could be found in plant organogenesis, where cytoskeletal responses to growth coordinate robust shape emergence. Mechanosensitive pathways (e.g. Hippo/YAP) and channels (e.g. Piezo) are potentially important factors. Also, redundant mechanisms likely exist to compensate for errors. It has recently been shown that during formation of the *Drosophila* embryonic heart, two spatially distinct adhesion molecules can each partially buffer the loss of the other to ensure robust cardiogenesis [7]. As the tools to image development at greater spatial and temporal resolution improve, new opportunities arise to explore the processes ensuring organs are constructed reliably.

## Can organogenesis be studied outside of the organism?

The accessibility of developmental systems, particularly for live imaging, presents a major challenge in deepening our understanding of morphogenesis. So, moving outside the organism can be helpful; amazing recent work using in vitro approaches have enabled insight into the timing and control of cell fate decision making [8] and the self-organisation of body axes [9]. However, the next step is to extend this to organ formation within constrained boundaries and a more realistic physical environment.

Another route for exploring organogenesis is to learn from non-model systems. The emergence of complex shapes in, for example, butterflies and dung beetles, due to a variety of factors, may provide novel insights into how morphogenesis is regulated.

Morphogenesis is an inherently mechanical process, and we need to incorporate biophysical thinking into our knowledge of the underlying genetics to understand how complex shapes emerge in development. Recent advances in mechanical models of biological systems (and the ability to test these in vivo) have also provided a framework for tackling morphogenesis. Many of the above questions require integrating spatial scales from the protein level up to the whole organism and timescales that span from milliseconds to days. Encapsulating such information within a manageable framework is challenging. However, this is the sort of problem tackled by physics. For example, modelling has been instrumental in understanding how the information encoded within morphogen gradients is translated precisely into gene expression boundaries [2], including in growing systems. The language of phase transitions (e.g. in liquid droplets) and emergent phenomena [10] will likely become more commonplace over the next few years in developmental biology.

## References

[1] Whitfield TT (2015). Development of the inner ear. Curr Opin Genet Dev.

[2] Zagorski M (2017). Decoding of position in the developing neural tube from antiparallel morphogen gradients. Science..

[3] Yu F-X (2015). Hippo pathway in organ size control, tissue homeostasis, and cancer. Cell..

[4] Cao J (2017). Tension creates an endoreplication wavefront that leads regeneration of epicardial tissue. Dev Cell.

[5] Droujinine IA, Perrimon N (2016). Interorgan communication pathways in physiology: focus on Drosophila. Annu Rev Genet.

[6] Mickoleit M (2014). High-resolution reconstruction of the beating zebrafish heart. Nat Methods.

[7] Zhang S (2018). Selective filopodia adhesion ensures robust cell matching in the Drosophila heart. Dev Cell.

[8] Sonnen KF (2018). Modulation of phase shift between Wnt and notch signaling oscillations controls mesoderm segmentation. Cell..

[9] Beccari L (2018). Multi-axial self-organization properties of mouse embryonic stem cells into gastruloids. Nature..

[10] Shin Y, Brangwynne CP (2017). Liquid phase condensation in cell physiology and disease. Science.

